# Foods, Nutrients, and Risk of In-Hospital Frailty in Women: Findings from a Large Prospective Cohort Study

**DOI:** 10.3390/nu15214619

**Published:** 2023-10-31

**Authors:** Huifeng Zhang, Weimin Li, Youfa Wang, Yuanyuan Dong, Darren C. Greenwood, Laura J. Hardie, Janet E. Cade

**Affiliations:** 1 Clinical Nutrition Department, The First Affiliated Hospital of Xi’an Jiaotong University, 277 Yanta West Road, Xi’an 710061, China; hfzhang@xjtu.edu.cn; 2 Nutritional Epidemiology Group, School of Food Science and Nutrition, University of Leeds, Leeds LS2 9JT, UK; fsydo@leeds.ac.uk (Y.D.); j.e.cade@leeds.ac.uk (J.E.C.); 3 Global Health Institute, School of Public Health, Xi’an Jiaotong University, Xi’an 710116, China; 4 Leeds Institute for Data Analytics, Faculty of Medicine and Health, University of Leeds, Leeds LS2 9JT, UK; d.c.greenwood@leeds.ac.uk; 5 Division of Clinical and Population Sciences, Leeds Institute of Cardiovascular and Metabolic Medicine, School of Medicine, University of Leeds, Leeds LS2 9JT, UK; l.j.hardie@leeds.ac.uk

**Keywords:** frailty, dietary intakes, meat consumption, processed meat, nutrients, hospital episode statistics

## Abstract

Frailty is increasingly prevalent worldwide because of aging populations. Diet may play a role as a modifiable risk factor. This study aimed to investigate associations between dietary factors and risk of frailty in the UK Women’s Cohort admitted to hospitals in England. Consumption of foods and nutrients was estimated using a validated 217-item food frequency questionnaire at baseline. Incident frailty was assessed via a hospital frailty risk score based on linkage with hospital episode statistics. Out of 25,186 participants admitted to hospitals, 6919 (27%) were identified with frailty and 10,562 (42%) with pre-frailty over a mean follow-up of 12.7 years. After adjustment for confounding, we observed a 12% increase in risk of frailty with each additional 10 g/MJ intake of total meat (HR = 1.12, 95%CI: 1.07, 1.17), with the highest risk observed for processed meats (HR = 1.45, 95%CI: 1.21, 1.73). Similar associations were observed with pre-frailty. Vegetable intake was associated with slightly lower risk of frailty (HR = 0.98, 95%CI: 0.97, 1.00). There was no evidence of association between most nutrient intakes and in-hospital frailty risk. Overall, our findings suggest that reducing consumption of meat, especially processed meat, in adults may be beneficial regarding the development of frailty.

## 1. Introduction

Frailty is a clinical syndrome characterized by cumulative loss of physiologic reserve and increased vulnerability to stressors. It is highly associated with increased risk of negative health-related outcomes, including falls, fractures, disability, and death [1]. The prevalence of frailty increases with age and rates are higher in women than in men [2,3]. Frailty is highly prevalent among hospital inpatients, with rates ranging from 4% to 89% in various settings [4]. A meta-analysis of over 450,000 geriatric hospital inpatients reported a prevalence of 47.4% for frailty and 73.2% for pre-frailty and frailty combined [5]. Frailty is an important risk factor for mortality and a broad range of adverse clinical outcomes, with the latter reflected in higher health-care use and expenditure [3,6,7]. It is reported that frail women have double the odds of hospitalization and three-fold increased odds of a nursing facility stay, and incur greater medical costs compared to robust women [8]. Given the health effects and rapidly growing aging population, frailty is increasingly gaining international attention as an important global health challenge [6].

A growing body of evidence points to a key role of nutrition in aging and age-related disease [9]. Dietary factors, encompassing dietary patterns, food groups, and intakes of calories, macro-nutrients, and micro-nutrients could be associated with the pathophysiology of frailty. Generally, healthier dietary patterns with high consumption of fruit, vegetables, and whole grains have been associated with lower frailty risk, both in cross-sectional and longitudinal studies [10]. However, significant heterogeneity is present across studies, and much of the evidence is either cross-sectional or limited in duration of follow-up. Although some longitudinal studies showed possible protective benefits from high consumption of fruit and vegetables [11,12] and low-fat dairy foods [13], and low consumption of ultra-processed foods [14], the number of studies is limited and the effects of these foods on the onset and progression of frailty are still unclear. There is a pressing need for better evidence to clarify potentially protective or harmful effects of food groups, nutrient intakes, and more on frailty risk.

As a modifiable lifestyle factor, nutrition could emerge as a potential target for future prevention and treatment strategies for frailty. This study aims to provide high-quality evidence for associations between diet and in-hospital frailty risk utilizing a large-scale population cohort, the UK Women’s Cohort, with an extended period of follow-up.

## 2. Materials and Methods

### 2.1. Study Population

The UK Women’s Cohort Study (UKWCS) has collected demographic, anthropometric, dietary, lifestyle, and health-related information from 35,372 women aged 35–69 years who responded to a postal questionnaire across England, Scotland, and Wales at baseline recruitment (1995 to 1998). The UKWCS was designed to explore potential associations between diet and chronic diseases, and has been described in detail elsewhere [15]. A subgroup of UKWCS participants who were admitted to hospitals in England were included in this study. A flow chart of UKWCS participants for this study is shown in Supplementary Figure S1.

At the cohort’s inception in 1993, ethical approval was obtained from National Research Ethics Service (NRES) Committee for Yorkshire & the Humber—Leeds East (Ref: 15/YH/0027), and approval was updated to include linkage outcomes and related sub-studies (Health Research Authority, REC Reference: 17/YH/0144), along with an NHS Digital Data Sharing Agreement (DARS-NIC-109867-M8S6B-v1.5) for the UKWCS-HES database to include matched medical records.

### 2.2. Dietary Assessment

Dietary information at baseline was obtained from a self-administered 217-food item food frequency questionnaire (FFQ), which was adapted from the FFQ for the European Prospective Investigation into Cancer and Nutrition (EPIC) study [16]. This FFQ has been validated against four-day weighed food diaries and a second FFQ collected at the same time as the diary, involving 283 women 3 years after baseline. Whilst accepting that each tool type measures different aspects of diet, correlations between the two dietary assessment methods were comparable to those found in other studies [17,18]. The daily consumed food weight of each item (g/d) was calculated from daily portions multiplied by standard portion weights from the Food Standards Agency portion sizes book [19]. Daily portions were converted from food intake frequencies in the FFQ (details in Supplementary Table S1). Further, daily intakes of energy, macro-nutrients, and micro-nutrients were calculated by summing nutrient contents of each food item, from the consumed food weights multiplied by the standard nutrient composition of foods derived from McCance and Widdowson’s The Composition of Foods (5th Edition) [20]. Nutrients provided by supplements were not included in this study.

In nutritional analyses of this study, the energy density method was used to model food and nutrient intakes [21]. Macro-nutrients (protein, carbohydrates, and fat) were included as the percentage of total energy derived from each one. Micro-nutrients and main foods were expressed as the intake per MJ of total energy. Then the multivariate energy density method was used in multiple regression models with daily energy intake as a covariate, as recommended by Willett et al. [21].

### 2.3. Outcome Variables

Incident cases of frailty were identified through a hospital frailty risk score (hFRS) developed and validated by Gilbert et al. [22]. Briefly, hFRS was based on International Classification of Diseases (ICD)-10 diagnostic codes that were the most related to frailty syndromes. The ICD-10 diagnostic codes in UKWCS came from linkage to hospital episode statistics (HES) of the UK National Health Service up to 31 March 2019. The HES contains multiple hospitalization records for each included participant with the main and secondary diagnostic ICD codes. The ICD codes listed in Supplementary Table S2 were assigned a corresponding point, while those not listed were given a point of 0. A hFRS for each participant was calculated by summing points from all diagnostic codes of one admitted hospital record. Participants were followed from study entry until first diagnosis of an event associated with frailty, date of death, or until the censor date (31 March 2019), whichever came first. Cases of pre-frailty or more severe frailty were defined if the hFRS > 0, and cases of frailty were defined if the hFRS ≥ 2.

### 2.4. Statistical Analysis

Descriptive statistics were used to summarize baseline socio-demographic, lifestyle, and nutritional characteristics for UKWCS participants within three groups of hFRS separately. Cox proportional hazards regression was used to estimate hazard ratios (HRs) and 95% confidence intervals (95% CIs) for associations between each dietary factor and risk of pre-frailty and frailty. For ease of interpretation, the HRs were presented per 10 g of the food groups per MJ of total energy consumed, as indicated in the results tables.

Unadjusted and multivariable-adjusted models were developed in this study. Potential risk factors for frailty previously identified in the literature were considered as covariates in the adjusted model, including age at baseline; ethnicity (white, Asian, black, and other); marital status (married/living as married, separated/divorced, and single/widowed); socio-economic status (SES, professional/managerial, intermediate, and routine/manual); physical activity (low, moderate, and high levels); smoking status (never smoked, ex-smoker, and current smoker); alcohol consumption (g/d, continuous); body mass index (BMI, kg/m^2^, continuous); and daily energy intake (MJ/d, continuous). Most variables were self-reported at recruitment. SES was derived from the United Kingdom National Statistics-Socio-Economic Classification (NS-SEC) [23]. Physical activity was calculated based on a series of questions about participants’ usual daily activities at baseline that were taken from the International Physical Activity Questionnaire (IPAQ) short form and categorized into three levels, being low, moderate, and high, according to the official guidelines for data processing and analysis [24].

To examine the effects relative to age on which frailty usually strongly depends, interaction terms between dietary factors and age were included in the Cox models, where age was used linearly. Additionally, subgroup analysis was conducted by fitting the models with participants <60 years old and ≥60 years old (at recruitment) separately. To further check for possible reverse causation, sensitivity analysis was conducted by excluding participants with survival time <3 years. A final sensitivity analysis was performed excluding participants aged <65 years at diagnosis to check for potential selection bias via inclusion of young age groups.

All statistical analyses were conducted using Stata/MP, version 17.0 (Stata Corp LP, College Station, TX, USA).

## 3. Results

### 3.1. Socioeconomic Characteristics and Dietary Intakes at Baseline

Of the 35,372 women at recruitment, 9980 women who did not have episode statistic hospital records and 206 women who had incomplete hospital records were excluded, leaving 25,186 women for analyses. Baseline characteristics of the participants admitted to hospitals are summarized according to hFRS levels in Table 1. After a mean follow-up of 12.7 years, there were 7705 women admitted to hospitals with no frailty (hFRS = 0), 10,562 women admitted to hospitals with pre-frailty (0 < hFRS < 2), and 6919 women admitted to hospitals with frailty (hFRS ≥ 2). On average, participants were 53.1 years old (standard deviation, SD = 9.4) at recruitment, and women with frailty or pre-frailty were older than women with no frailty. Consistent with this, women with frailty had an older mean age at diagnosis (64.1 years) compared with the other two groups (58.9 years hFRS = 0, 61.5 years 0 < hFRS < 2). Compared with participants with no frailty, those with frailty or pre-frailty had a higher proportion of no qualifications, were more likely to be single or widowed, and less likely to have professional or managerial jobs. The proportions of different ethnicity and levels of physical activity were similar across three groups. Body mass index was higher in women with pre-frailty (mean (SD) 24.8 (4.4) kg/m^2^) and frailty (24.9 (4.5) kg/m^2^) than women with no frailty (24.1 (3.9) kg/m^2^). Women with no frailty reported drinking slightly more alcohol and smoking less than the other groups.

Profiles of consumed main foods among participants admitted to hospitals in the UKWCS are summarized by frailty status in Table 2. Women with frailty consumed the highest absolute total fish, processed meat, red meat, and total meat, whilst women with no frailty consumed the lowest. Consumption of vegetables, fruits, and poultry was generally similar across the three groups. Dietary intakes of energy and nutrients in each group are summarized in Supplementary Table S3. Generally, there was little difference in daily intakes of energy and most nutrients at baseline across the three groups.

### 3.2. Associations between Dietary Intakes and In-Hospital Frailty Risk

**Figure 1:**
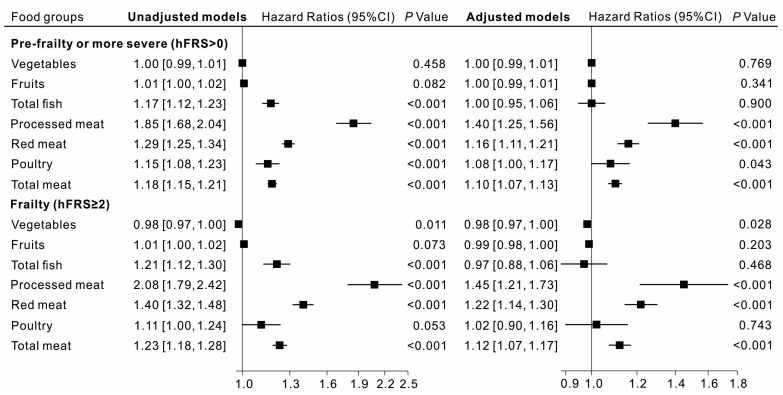
Associations between food groups (per 10 g/MJ) and in-hospital frailty risk within the UK Women’s Cohort Study. Age, ethnicity, marital status, socioeconomic status, physical activity, body mass index, smoking status, alcohol consumption, and total energy intake were adjusted in the adjusted model in the right panel of the figure; 95%CI, 95% confidence interval; hFRS, hospital frailty risk score.

As shown in the upper panel of Figure 1, after adjustment for potential confounders, risk of pre- and more severe frailty (hFRS > 0) was 40% higher (HR = 1.40, 95%CI: 1.25, 1.56) with every additional 10 g/MJ of processed meat, 16% higher (HR = 1.16, 95%CI: 1.11, 1.21) per 10 g/MJ red meat, 8% higher (HR = 1.08, 95%CI: 1.00, 1.17) per 10 g/MJ poultry, and 10% higher (HR = 1.10, 95%CI: 1.07, 1.13) per 10 g/MJ total meat.

Similarly, for frailty (hFRS ≥ 2), as shown in the lower panel of Figure 1, risk was 45% higher (HR = 1.45, 95%CI: 1.21, 1.73) per 10 g/MJ processed meat, 22% higher (HR = 1.22, 95%CI: 1.14, 1.30) per 10 g/MJ red meat, 2% higher (HR = 1.02, 95%CI: 0.90, 1.16) per 10 g/MJ poultry, and 12% higher (HR = 1.12, 95%CI: 1.07, 1.17) per 10 g/MJ total meat.

Vegetable intake was associated with slightly lowered risk of frailty (hFRS ≥ 2) (HR = 0.98, 95%CI: 0.97, 1.00), but there was insufficient evidence of any association with risk of pre-frailty or more severe frailty (hFRS > 0) (HR = 1.00, 95%CI: 0.99, 1.01). There was no evidence of any association between consumption of fruits and total fish with risk of either pre-frailty or frailty.

Associations between intakes of energy and nutrients and in-hospital risk of pre-frailty or frailty are shown in the Supplementary Table S4. Daily intakes of vitamin B12 and zinc were associated with increased risk of pre-frailty or more severe frailty by 11% (HR = 1.11, 95%CI: 1.05, 1.17) and 12% (HR = 1.12, 95%CI: 1.04, 1.21), respectively, in adjusted models. All other nutrient intakes were not observed to be associated with frailty risk.

### 3.3. Subgroup Analysis and Sensitivity Analysis

For subgroup analysis in Table 3, the in-hospital risk of pre- and more severe frailty associated with consumption of processed meat, red meat, and total meat was higher in participants with age ≥60 years old compared to those with age <60 years old, where *p*-values for the interaction effect between age and each dietary factor were significant (0.015, 0.001, and <0.001 respectively). Similarly, the risk of frailty in relation to consumption of poultry and total meat was higher in participants with age ≥60 years old compared to those with age <60 years old, where *p*-interaction was 0.027 and 0.009, respectively. There was no significant evidence on effect modification of age on the remaining food groups (Table 3) and most nutrients (Supplementary Tables S5 and S6), except that a higher risk of frailty was observed in subjects with age ≥60 years old than those with age <60 years old associated with daily intake of zinc (*p*-interaction = 0.007 in Supplementary Table S5).

In a sensitivity analysis, 3052 participants with a survival time <3 years were excluded to check for possible reverse causation. The risk of pre-frailty or frailty in relation to dietary factors appeared slightly attenuated, but did not change substantially after excluding those individuals (Supplementary Tables S7 and S8). In another sensitivity analysis, results were robust where participants (n = 15,601) aged <65 years at diagnosis were excluded (data shown in Supplementary Tables S9 and S10).

## 4. Discussion

This study found a significantly higher in-hospital risk of prefrailty or frailty associated with consumption of processed meat, red meat, and total meat. Subgroup analysis showed increased magnitude of these associations among individuals aged ≥60 years old compared to those <60 years old at baseline. Sensitivity analyses showed results were robust to the removal of participants with survival times <3 years in adjusted models.

Consumption of meat, especially red meat rich in pro-oxidative iron, is considered as part of a pro-inflammatory diet [25]. Analysis of a large, population-based cohort study of 455,776 participants in UK Biobank reported that a meat-based diet characterized by high consumption of red meat (lamb, pork, and beef) and processed meat was positively correlated with several pro-inflammatory biomarkers, including key leukocytes, C-reactive protein, and an aggregated inflammation score [26]. Recent studies have suggested a pro-inflammatory diet may be associated with increased risk of frailty [27,28]. A pro-inflammatory mechanism may provide a rationale for the positive association detected in this study between meat consumption and risk of frailty. Prospective evidence of frailty risk in relation to meat consumption is limited. Most previous studies tend to support a high intake of protein is recommended for the elderly to prevent frailty risk [29,30]. Although red meat and processed meat is rich in protein, we speculate that pro-inflammatory factors such as iron containing heme, saturated fat, and high levels of nitrates, nitrites, and amines may offset the protective effect of protein from meat. However, our findings need to be confirmed in other large longitudinal studies.

Most nutrient intakes were not observed to be associated with risk of frailty in our study and not consistent with mainstream opinion that protein supplementation, combined with physical activity, are an effective way to prevent physical frailty in elderly people [31,32]. We speculate a key reason for the inconsistency includes heterogeneity of frailty assessment tools. Previous studies have commonly used frailty tools based on phenotypes or deficits mainly including weight loss, exhaustion, slow gait speed, and weak grip strength [1], while our study used a hospital frailty score based on ICD diagnostic codes related to frailty syndromes, where frailty status is more likely to be severe. Heterogeneous assessment tools could assess different aspects of frailty, which may in turn modify the dietary associations detected. Intakes of vitamin B12 and zinc were found to be positively associated with risk of pre-frailty and frailty in our study. High levels of vitamin B12 are found to be associated with negative effects, such as inflammation and poor outcome for critically ill patients [33]. As reviewed, both deficient and high levels of vitamin B12 are risk factors for various clinical morbidities, and its levels potentially have an impact on frailty [34]. At present, there is little evidence on associations between intakes of zinc and risk of frailty. Generally, associations between nutrient intakes and frailty risk remain unconfirmed.

Currently, there is no uniform definition or assessment for the frailty complex. More than 60 assessment tools for frailty have been identified in scientific publications, of which nine are highly cited (≥200 citations), including the Fried Frailty Phenotype and the Rockwood Frailty Index [35]. The former, introduced by Fried et al., mainly comprises five phenotypes (weight loss, weakness, poor energy, slowness, low physical activity), where subjects having three or more phenotypes can be identified as frail [36]. The Rockwood Frailty Index is a broader deficit accumulation index, reflecting the proportion of potential deficits present in one person out of all considered deficits, including frailty symptoms, signs, diseases, and disabilities [37]. The hFRS tool has been validated against the two standard tests above and was used to assess the status of frailty in a hospital setting in this study [22]. Although most frailty assessment tools are more suitable for the elderly, our results were robust in sensitivity analysis to the exclusion of participants aged <65 years at diagnosis, indicating that the main results were not influenced substantially by inclusion of young participants in this study.

Strengths of this study include a large sample size and a longitudinal study design with relatively long follow-up time. In our study, the frailty assessment based on a cumulative score related to ICD codes from hospital records ensured identification accuracy and reduced reporting errors, as well as potential loss to follow-up over a long follow-up period. In addition, a variety of confounders including sociodemographic and lifestyle factors were adjusted for in our Cox proportional regression models. However, as for all observational studies, residual confounding is still possible. Moreover, as an observational study, causality cannot be established, although no obvious reverse causation was found in the sensitivity analysis of this study. In addition, taking hospital admission dates as a proxy for diagnosis dates of incident frailty could have resulted in measurement errors. Our study is also limited because only women admitted to hospitals in England were included in the analyses, which limits the generalizability of our findings; thus, more research is needed to investigate the risk of frailty in other populations.

In conclusion, our study revealed a link between in-hospital frailty risk and consumption of processed meat, red meat, and total meat. Further research is needed to elucidate the role of nutrition in strategies to reduce frailty. In particular, randomized controlled trials of plant-based protein as a meat substitute should be considered to provide high-quality evidence to support public health recommendations for preventing frailty.

## Figures and Tables

**Table 1:** Demographic characteristics of participants with different frailty scores at follow up in the UK Women’s Cohort Study.

		hFRS = 0 (N = 7705, 30.6%)	0 < hFRS < 2 (N = 10,562, 41.9%)	hFRS ≥ 2 (N = 6919, 27.5%)	p *	All Participants (N = 25,186)
Age at baseline (years)	Mean (standard deviation)	49.4 (8.1)	54.0 (9.4)	56.0 (9.7)	<0.001	53.1 (9.4)
Age at diagnosis (years)	Mean (standard deviation)	58.9 (9.9)	61.5 (10.2)	64.1 (10.7)	<0.001	61.4 (10.5)
Follow-up time (years)	Mean (standard deviation)	14.4 (6.3)	11.7 (6.2)	12.3 (6.2)	<0.001	12.7 (6.3)
Ethnicity (N, %)	White	7447 (98.6%)	10,104 (98.5%)	6615 (98.8%)	0.346	24,166 (98.6%)
	Asian	45 (0.6%)	72 (0.7%)	38 (0.6%)		155 (0.6%)
	Black	13 (0.2%)	16 (0.2%)	11 (0.2%)		40 (0.2%)
	other	49 (0.7%)	66 (0.6%)	29 (0.4%)		144 (0.6%)
Educational level (N, %)	No qualifications	854 (11.8%)	1974 (20.8%)	1456 (23.8%)	<0.001	4284 (18.8%)
	O-level or equivalent	2404 (33.3%)	3097 (32.6%)	1796 (29.4%)		7297 (31.9%)
	A-level or equivalent	1825 (25.3%)	2184 (23.0%)	1409 (23.1%)		5418 (23.7%)
	University degree	2137 (29.6%)	2255 (23.7%)	1452 (23.8%)		5844 (25.6%)
Marital status (N, %)	Married or living as married	6023 (79.1%)	7675 (73.9%)	4890 (71.8%)	<0.001	18,588 (74.9%)
	Separated or divorced	804 (10.6%)	1158 (11.2%)	760 (11.2%)		2722 (11.0%)
	Single or widowed	787 (10.3%)	1556 (15.0%)	1162 (17.1%)		3505 (14.1%)
Socio-economic status (SES) (N, %)	Routine and manual	599 (7.9%)	1073 (10.4%)	707 (10.5%)	<0.001	2379 (9.7%)
	Intermediate	2086 (27.5%)	2912 (28.2%)	1984 (29.4%)		6982 (28.3%)
	Professional and managerial	4902 (64.6%)	6329 (61.4%)	4053 (60.1%)		15,284 (62.0%)
Physical activity	Low level	791 (10.3%)	1211 (11.5%)	791 (11.4%)	0.178	2793 (11.1%)
(N, %)	Moderate level	3918 (50.9%)	5205 (49.3%)	3379 (48.8%)		12,502 (49.6%)
	High level	2996 (38.9%)	4146 (39.3%)	2749 (39.7%)		9891 (39.3%)
Body mass index (BMI) (kg/m^2^)	Mean (standard deviation)	24.1 (3.9)	24.8 (4.4)	24.9 (4.5)	<0.001	24.6 (4.3)
Alcohol (g/d)	Mean (standard deviation)	9.2 (10.3)	8.4 (10.5)	8.2 (10.0)	<0.001	8.6 (10.3)
Smoking status (N, %)	Never smoked	4477 (59.6%)	5707 (55.7%)	3745 (56.1%)	<0.001	13,929 (57.0%)
	Ex-smoker	2249 (30.0%)	3318 (32.4%)	2184 (32.7%)		7751 (31.7%)
	Current smoker	783 (10.4%)	1218 (11.9%)	742 (11.1%)		2743 (11.2%)

* Difference was tested using Student’s *t*-test for continuous variables and χ2 test for categorical variables; hFRS, hospital frailty risk score.

**Table 2:** Profiles of consumed main foods (g/day) among participants with different frailty scores at follow up in the UK Women’s Cohort Study.

Food Groups	hFRS = 0 (N = 7705, 30.6%)	0 < hFRS < 2 (N = 10,562, 41.9%)	hFRS ≥ 2 (N = 6919, 27.5%)	p *	All Participants (N = 25,186)
Vegetables	316 (183)	322 (206)	318 (197)	0.120	319 (196)
Fruits	306 (226)	319 (256)	323 (251)	0.152	316 (246)
Total fish	27 (25)	29 (31)	30 (27)	<0.001	29 (28)
Processed meat	12 (14)	13 (16)	14 (16)	<0.001	13 (15)
Red meat	32 (38)	35 (44)	37 (47)	<0.001	34 (43)
Poultry	17 (20)	17 (21)	17 (22)	0.141	17 (21)
Total meat	63 (61)	67 (69)	70 (71)	<0.001	67 (67)

* Difference was tested by Student’s *t*-test; hFRS, hospital frailty risk score.

**Table 3:** Subgroup analysis by age on associations between food groups (per 10 g/MJ) and in-hospital frailty risk within the UK Women’s Cohort Study.

	Hazard Ratio (95% Confidence Interval)				
	<60 Years Old		≥60 Years Old		** p-Interaction With Age
	Adjusted *	p *	Adjusted *	p *	
**Risk of Pre- and more severe frailty (hFRS > 0)**					
Vegetables	1.01 (0.99, 1.02)	0.383	0.99 (0.97, 1.01)	0.196	0.052
Fruits	1.00 (0.99, 1.01)	0.740	0.99 (0.97, 1.00)	0.083	0.154
Total fish	1.02 (0.95, 1.09)	0.614	0.99 (0.90, 1.09)	0.882	0.567
Processed meat	1.36 (1.18, 1.56)	<0.001	1.50 (1.23, 1.83)	<0.001	0.015
Red meat	1.14 (1.09, 1.21)	<0.001	1.19 (1.10, 1.28)	<0.001	0.001
Poultry	1.10 (1.00, 1.21)	0.042	1.07 (0.94, 1.22)	0.318	0.028
Total meat	1.09 (1.06, 1.13)	<0.001	1.13 (1.08, 1.19)	<0.001	<0.001
**Risk of Frailty (hFRS ≥ 2)**					
Vegetables	0.99 (0.98, 1.01)	0.591	0.97 (0.95, 0.99)	0.016	0.135
Fruits	1.00 (0.99, 1.02)	0.818	0.98 (0.96, 1.00)	0.038	0.708
Total fish	1.02 (0.91, 1.15)	0.681	0.90 (0.78, 1.04)	0.141	0.791
Processed meat	1.43 (1.14, 1.80)	0.002	1.54 (1.15, 2.06)	0.004	0.185
Red meat	1.24 (1.14, 1.35)	<0.001	1.20 (1.08, 1.34)	0.001	0.115
Poultry	1.01 (0.87, 1.19)	0.855	1.06 (0.87, 1.29)	0.586	0.027
Total meat	1.12 (1.06, 1.19)	<0.001	1.14 (1.05, 1.23)	0.001	0.009

hFRS, hospital frailty risk score; * adjusted for age, ethnicity, marital status, socioeconomic status, physical activity, body mass index, smoking status, alcohol consumption, and total energy intake; ** *p*-interaction represents the statistical significance for interaction item of dietary factor and age where age was modelled linearly in the Cox proportional regression.

## Data Availability

Data is unavailable due to participants’ privacy and ethical restrictions.

## Supplementary Materials

The following supporting information can be downloaded at: https://www.mdpi.com/article/10.3390/nu15214619/s1, Table S1: Transformation from intake frequencies to portion servings per day; Table S2: Frailty-related ICD-10 diagnostic codes derived from Gilbert et al. [22]; Table S3: Profiles of nutrient intakes by frailty status within the UK Women’s Cohort Study; Table S4: Associations of nutrient intakes with in-hospital risk of pre-frailty and frailty within the UK Women’s Cohort Study; Table S5: Subgroup analysis by age on associations between nutrient intakes and in-hospital risk of pre-frailty or more severe frailty within the UK Women’s Cohort Study; Table S6: Subgroup analysis by age on associations between nutrient intakes and in-hospital risk of frailty within the UK Women’s Cohort Study; Table S7: Sensitivity analysis to check for reverse causation on main foods within the UK Women’s Cohort Study; Table S8: Sensitivity analysis to check for reverse causation on nutrient intakes within the UK Women’s Cohort Study; Table S9: Sensitivity analysis excluding participants aged <65 y at diagnosis on main foods (per 10 g/MJ) within the UK Women’s Cohort Study; Table S10: Sensitivity analysis excluding participants aged <65 y at diagnosis on nutrient intakes within the UK Women’s Cohort Study.
