# Supplementary material for: Foods, Nutrients, and Risk of In-Hospital Frailty in Women: Findings from a Large Prospective Cohort Study
Source: Nutrients. 2023 Oct 31;15(21):4619. doi: 10.3390/nu15214619 (PMC10650049; doi:10.3390/nu15214619)
Supplement: Supplementary file 1 [file nutrients-15-04619-s001.zip › nutrients-2639636-supplementary.pdf]

Foods, nutrients and risk of in-hospital frailty in women: findings from a large prospective cohort study

Supplementary Methods

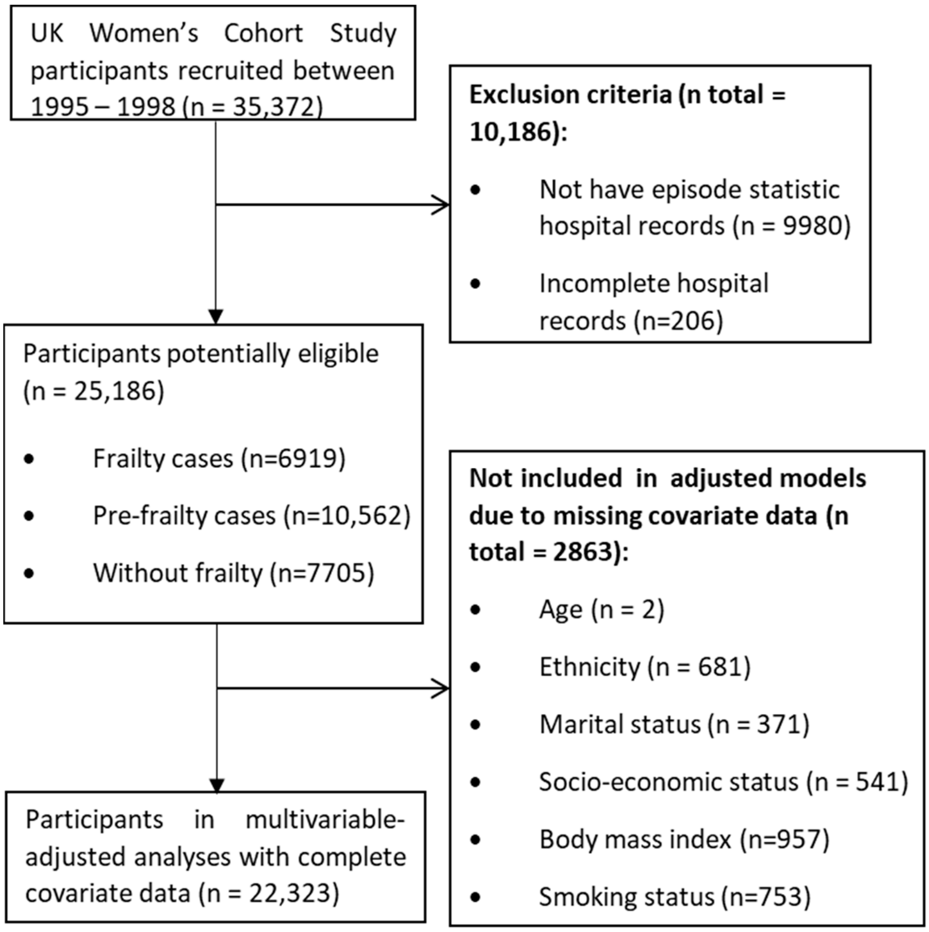

Supplementary Figure S1 The flow chart of UKWCS participants for this study

Supplementary Table S2 Transformation from intake frequencies to portion servings per day

Frequency of intake	Portion servings per day (formula)
Never	0
Less than once a month	0.02 (0.5/30)
1-3 per month	0.07 (2.0/30)
Once a week	0.14 (1.0/7)
2-4 per week	0.40 (3.0/7)
5-6 per week	0.80 (5.5/7)
Once per day	1.00 (1.0/1)
2-3 per day	2.50 (2.5/1)
4-5 per day	4.50 (4.5/1)
6+ per day	6.00 (6.0/1)

Supplementary Table S2 Frailty related ICD-10 diagnostic codes derived from Gilbert *et al.* [1]

ICD Code	ICD Description	Points awarded	ICD Code	ICD Description	Points awarded
F00	Dementia in Alzheimer's disease	7·1	E16	Other disorders of pancreatic internal secretion	1·4
G81	Hemiplegia	4·4	R94	Abnormal results of function studies	1·4
G30	Alzheimer's disease	4·0	N18	Chronic renal failure	1·4
I69	Sequelae of cerebrovascular disease	3·7	R33	Retention of urine	1·3
R29	Other symptoms and signs involving the nervous and musculoskeletal systems	3·6	R69	Unknown and unspecified causes of morbidity	1·3
N39	Other disorders of urinary system (includes urinary tract infection and urinary incontinence)	3·2	N28	Other disorders of kidney and ureter, not elsewhere classified	1·3
F05	Delirium, not induced by alcohol and other psychoactive substances	3·2	R32	Unspecified urinary incontinence	1·2
W19	Unspecified fall	3·2	G31	Other degenerative diseases of nervous system, not elsewhere classified	1·2
S00	Superficial injury of head	3·2	Y95	Nosocomial condition	1·2
R31	Unspecified haematuria	3·0	S09	Other and unspecified injuries of head	1·2
B96	"Other bacterial agents as the cause of diseases classified to other chapters"	2·9	R45	Symptoms and signs involving emotional state	1·2
R41	"Other symptoms and signs involving cognitive functions and awareness"	2·7	G45	Transient cerebral ischaemic attacks and related syndromes	1·2
R26	Abnormalities of gait and mobility	2·6	Z74	Problems related to care-provider dependency	1·1
I67	Other cerebrovascular diseases	2·6	M79	Other soft tissue disorders, not elsewhere classified	1·1
R56	Convulsions, not elsewhere classified	2·6	W06	Fall involving bed	1·1
R40	Somnolence, stupor and coma	2·5	S01	Open wound of head	1·1
T83	"Complications of genitourinary prosthetic devices, implants and grafts"	2·4	A04	Other bacterial intestinal infections	1·1
S06	Intracranial injury	2·4	A09	Diarrhoea and gastroenteritis of presumed infectious origin	1·1
S42	Fracture of shoulder and upper arm	2·3	J18	Pneumonia, organism unspecified	1·1
E87	"Other disorders of fluid, electrolyte and acid base balance"	2·3	J69	Pneumonitis due to solids and liquids	1·0
M25	Other joint disorders, not elsewhere classified	2·3	R47	Speech disturbances, not elsewhere classified	1·0
E86	Volume depletion	2·3	E55	Vitamin D deficiency	1·0
R54	Senility	2·2	Z93	Artificial opening status	1·0
Z50	"Care involving use of rehabilitation procedures"	2·1	R02	Gangrene, not elsewhere classified	1·0
F03	Unspecified dementia	2·1	R63	Symptoms and signs concerning food and fluid intake	0·9
W18	Other fall on same level	2·1	H91	Other hearing loss	0·9
Z75	"Problems related to medical facilities and other health care"	2·0	W10	Fall on and from stairs and steps	0·9
F01	Vascular dementia	2·0	W01	Fall on same level from slipping, tripping and stumbling	0·9
S80	Superficial injury of lower leg	2·0	E05	Thyrotoxicosis [hyperthyroidism]	0·9
L03	Cellulitis	2·0	M41	Scoliosis	0·9
H54	Blindness and low vision	1·9	R13	Dysphagia	0·8
E53	Deficiency of other B group vitamins	1·9	Z99	Dependence on enabling machines and devices	0·8
Z60	Problems related to social environment	1·8	U80	Agent resistant to penicillin and related antibiotics	0·8
G20	Parkinson's disease	1·8	M80	Osteoporosis with pathological fracture	0·8
R55	Syncope and collapse	1·8	K92	Other diseases of digestive system	0·8
S22	Fracture of rib(s), sternum and thoracic spine	1·8	I63	Cerebral Infarction	0·8
K59	Other functional intestinal disorders	1·8	N20	Calculus of kidney and ureter	0·7
N17	Acute renal failure	1·8	F10	Mental and behavioural disorders due to use of alcohol	0·7
L89	Decubitus ulcer	1·7	Y84	Other medical procedures as the cause of abnormal reaction of the patient	0·7
Z22	Carrier of infectious disease	1·7	R00	Abnormalities of heart beat	0·7

B95	Streptococcus and staphylococcus as the cause of diseases classified to other chapters	1·7	J22	Unspecified acute lower respiratory infection	0·7
L97	Ulcer of lower limb, not elsewhere classified	1·6	Z73	Problems related to life-management difficulty	0·6
R44	Other symptoms and signs involving general sensations and perceptions	1·6	R79	Other abnormal findings of blood chemistry	0·6
K26	Duodenal ulcer	1·6	Z91	Personal history of risk-factors, not elsewhere classified	0·5
I95	Hypotension	1·6	S51	Open wound of forearm	0·5
N19	Unspecified renal failure	1·6	F32	Depressive episode	0·5
A41	Other septicaemia	1·6	M48	Spinal stenosis (secondary code only)	0·5
Z87	Personal history of other diseases and conditions	1·5	E83	Disorders of mineral metabolism	0·4
J96	Respiratory failure, not elsewhere classified	1·5	M15	Polyarthrosis	0·4
X59	Exposure to unspecified factor	1·5	D64	Other anaemias	0·4
M19	Other arthrosis	1·5	L08	Other local infections of skin and subcutaneous tissue	0·4
G40	Epilepsy	1·5	R11	Nausea and vomiting	0·3
M81	Osteoporosis without pathological fracture	1·4	K52	Other noninfective gastroenteritis and colitis	0·3
S72	Fracture of femur	1·4	R50	Fever of unknown origin	0·1
S32	Fracture of lumbar spine and pelvis	1·4			

[1] Gilbert, T., et al., Development and validation of a Hospital Frailty Risk Score focusing on older people in acute care settings using electronic hospital records: an observational study. The Lancet, 2018. **391**(10132): p. 1775-1782.

Supplementary Results

Supplementary Table S3 Profiles of nutrient intakes by frailty score levels within the UK Women's Cohort Study

		**hFRS=0 (N=7705, 30.6%)**	**0< hFRS <2 (N=10,562, 41.9%)**	**hFRS ≥2 (N=6919, 27.5%)**	***P*** *	**All participants (N=25,186)**
Energy intake	(kcal/day)	2343 (712)	2340 (840)	2358 (795)	0.289	2346 (790)
	(MJ/day)	10 (3)	10 (4)	10 (3)	0.289	10 (3)
Protein	(g/day)	89 (27)	90 (34)	91 (32)	<0.001	90 (32)
	(%energy)	15 (3)	16 (3)	16 (3)	<0.001	16 (3)
Carbohydrate	(g/day)	311 (103)	312 (116)	314 (113)	0.204	312 (111)
	(%energy)	53 (7)	53 (7)	53 (7)	0.030	53 (7)
Fat	(g/day)	85 (32)	84 (38)	85 (36)	0.162	85 (36)
	(%energy)	32 (6)	32 (6)	32 (6)	0.013	32 (6)
SFAs	(g/day)	29 (13)	29 (15)	30 (14)	0.040	29 (14)
	(%energy)	11 (3)	11 (3)	11 (3)	0.020	11 (3)
PUFAs	(g/day)	16 (7)	16 (8)	16 (8)	0.065	16 (8)
	(%energy)	6 (2)	6 (2)	6 (2)	<0.001	6 (2)
MUFAs	(g/day)	28 (11)	28 (13)	28 (13)	0.109	28 (12)
	(%energy)	11 (2)	10 (2)	11 (2)	0.001	11 (2)
Vitamin C	(mg/MJ)	18 (7)	18 (8)	18 (8)	0.001	18 (8)
Vitamin B1	(ug/MJ)	324 (253)	319 (245)	310 (245)	0.004	318 (247)
Vitamin B2	(ug/MJ)	263 (67)	270 (69)	268 (69)	<0.001	267 (68)
Vitamin B6	(ug/MJ)	286 (57)	292 (59)	290 (60)	<0.001	290 (58)
Vitamin B12	(ug/MJ)	0.58 (0.29)	0.62 (0.31)	0.62 (0.30)	<0.001	0.61 (0.30)
Folate	(ug/MJ)	41 (9)	42 (10)	42 (10)	<0.001	42 (10)
Vitamin A	(ug/MJ)	103 (53)	109 (58)	110 (56)	<0.001	108 (56)
Vitamin D	(ug/MJ)	0.31 (0.15)	0.33 (0.16)	0.33 (0.16)	<0.001	0.33 (0.16)
Vitamin E	(ug/MJ)	990 (300)	982 (304)	975 (308)	0.016	983 (304)
Calcium	(mg/MJ)	119 (28)	121 (29)	119 (29)	<0.001	120 (29)
Iron	(mg/MJ)	1.86 (0.56)	1.88 (0.58)	1.87 (0.58)	0.175	1.87 (0.57)
Zinc	(mg/MJ)	1.17 (0.21)	1.19 (0.22)	1.19 (0.22)	<0.001	1.18 (0.22)

* Difference was tested by Student's t test. SFAs, saturated fatty acids; PUFAs, polyunsaturated fatty acids; MUFAs, monounsaturated fatty acids.

Supplementary Table S4 Associations of nutrient intakes with in-hospital risk of prefrailty and frailty within the UK Women's Cohort Study

		Risk of Pre-Frailty or more severe (HR, 95%CI)				Risk of Frailty (HR, 95%CI)			
		Unadjusted	*P*	Adjusted*	*P**	Unadjusted	*P*	Adjusted*	*P**
Energy intake	per MJ	1.00 (1.00, 1.01)	0.126	1.01 (1.00, 1.01)	0.003	1.01 (1.00, 1.02)	0.023	1.01 (1.01, 1.02)	0.001
Protein	per %energy	1.03 (1.03, 1.04)	<0.001	1.01 (1.01, 1.02)	<0.001	1.03 (1.02, 1.04)	<0.001	1.00 (0.99, 1.01)	0.386
Carbohydrate	per %energy	1.00 (1.00, 1.00)	0.927	1.00 (1.00, 1.00)	0.142	1.00 (1.00, 1.00)	0.549	1.00 (0.99, 1.00)	0.049
Fat	per %energy	1.00 (1.00, 1.00)	0.213	1.00 (1.00, 1.00)	0.639	1.00 (1.00, 1.00)	0.700	1.00 (1.00, 1.01)	0.290
SFAs	per %energy	1.01 (1.00, 1.01)	<0.001	1.00 (1.00, 1.01)	0.259	1.02 (1.01, 1.03)	<0.001	1.01 (1.00, 1.02)	0.024
PUFAs	per %energy	0.97 (0.96, 0.98)	<0.001	1.00 (0.99, 1.01)	0.437	0.95 (0.94, 0.97)	<0.001	0.99 (0.98, 1.01)	0.385
MUFAs	per %energy	0.99 (0.99, 1.00)	0.025	1.00 (0.99, 1.01)	0.697	1.00 (0.99, 1.01)	0.638	1.01 (0.99, 1.02)	0.279
Vitamin C	per mg/MJ	1.00 (1.00, 1.00)	0.202	1.00 (1.00, 1.00)	0.759	1.00 (1.00, 1.00)	0.906	1.00 (0.99, 1.00)	0.085
Vitamin B1	per µg/MJ	1.00 (1.00, 1.00)	<0.001	1.00 (1.00, 1.00)	0.998	1.00 (1.00, 1.00)	<0.001	1.00 (1.00, 1.00)	0.317
Vitamin B2	per µg/MJ	1.00 (1.00, 1.00)	<0.001	1.00 (1.00, 1.00)	0.258	1.00 (1.00, 1.00)	0.004	1.00 (1.00, 1.00)	0.058
Vitamin B6	per µg/MJ	1.00 (1.00, 1.00)	<0.001	1.00 (1.00, 1.00)	0.002	1.00 (1.00, 1.00)	<0.001	1.00 (1.00, 1.00)	0.694
Vitamin B12	per µg/MJ	1.36 (1.30, 1.43)	<0.001	1.11 (1.05, 1.17)	<0.001	1.39 (1.29, 1.50)	<0.001	1.04 (0.96, 1.13)	0.361
Folate	per µg/MJ	1.00 (1.00, 1.00)	<0.001	1.00 (1.00, 1.00)	0.365	1.00 (1.00, 1.00)	0.071	1.00 (1.00, 1.00)	0.291
Vitamin A	per µg/MJ	1.00 (1.00, 1.00)	<0.001	1.00 (1.00, 1.00)	0.011	1.00 (1.00, 1.00)	<0.001	1.00 (1.00, 1.00)	0.880
Vitamin D	per µg/MJ	1.37 (1.26, 1.49)	<0.001	1.08 (0.98, 1.19)	0.105	1.42 (1.25, 1.61)	<0.001	1.00 (0.86, 1.17)	0.952
Vitamin E	per µg/MJ	1.00 (1.00, 1.00)	<0.001	1.00 (1.00, 1.00)	0.178	1.00 (1.00, 1.00)	<0.001	1.00 (1.00, 1.00)	0.178
Calcium	per mg/MJ	1.00 (1.00, 1.00)	0.057	1.00 (1.00, 1.00)	0.403	1.00 (1.00, 1.00)	0.162	1.00 (1.00, 1.00)	<0.001
Iron	per mg/MJ	1.00 (0.97, 1.03)	0.929	0.98 (0.95, 1.01)	0.142	0.98 (0.94, 1.02)	0.379	0.95 (0.90, 0.99)	0.016
Zinc	per mg/MJ	1.42 (1.33, 1.52)	<0.001	1.12 (1.04, 1.21)	0.003	1.45 (1.30, 1.62)	<0.001	1.04 (0.92, 1.18)	0.527

HR, Hazards Ratio; 95%CI, 95% Confidence Interval; SFAs, saturated fatty acids; PUFAs, polyunsaturated fatty acids; MUFAs, monounsaturated fatty acids.

* Adjusted for age, ethnicity, marital status, socioeconomic status, physical activity, body mass index, smoking status, alcohol consumption, and total energy intake

Supplementary Table S5 Subgroup analysis by age on associations between nutrient intakes and in-hospital risk of pre-frailty or more severe within the UK Women's Cohort Study

		Risk of Pre-frailty or more severe (Hazard Ratio, 95%CI)				
		<60 years old		≥60 years old		**P-interaction with age
		Adjusted*	P*	Adjusted*	P*	
Energy intake	per MJ	1.01 (1.00, 1.01)	0.057	1.01 (1.00, 1.02)	0.080	0.778
Protein	per %energy	1.01 (1.00, 1.02)	0.002	1.02 (1.00, 1.03)	0.005	0.020
Carbohydrate	per %energy	1.00 (1.00, 1.00)	0.479	1.00 (0.99, 1.00)	0.213	0.075
Fat	per %energy	1.00 (0.99, 1.00)	0.347	1.00 (0.99, 1.00)	0.833	0.504
SFAs	per %energy	1.00 (1.00, 1.01)	0.532	1.00 (0.99, 1.01)	0.721	0.420
PUFAs	per %energy	0.99 (0.98, 1.01)	0.311	1.00 (0.98, 1.02)	0.966	0.456
MUFAs	per %energy	1.00 (0.99, 1.01)	0.465	1.00 (0.99, 1.01)	0.895	0.494
Vitamin C	per mg/MJ	1.00 (1.00, 1.00)	0.382	1.00 (0.99, 1.00)	0.172	0.331
Vitamin B1	per µg/MJ	1.00 (1.00, 1.00)	0.664	1.00 (1.00, 1.00)	0.289	0.001
Vitamin B2	per µg/MJ	1.00 (1.00, 1.00)	0.230	1.00 (1.00, 1.00)	0.300	0.327
Vitamin B6	per µg/MJ	1.00 (1.00, 1.00)	0.003	1.00 (1.00, 1.00)	0.066	0.314
Vitamin B12	per µg/MJ	1.14 (1.06, 1.22)	<0.001	1.10 (1.01, 1.21)	0.033	0.336
Folate	per µg/MJ	1.00 (1.00, 1.00)	0.309	1.00 (1.00, 1.00)	0.601	0.897
Vitamin A	per µg/MJ	1.00 (1.00, 1.00)	0.001	1.00 (1.00, 1.00)	0.664	0.551
Vitamin D	per µg/MJ	1.05 (0.93, 1.18)	0.472	1.16 (1.00, 1.35)	0.045	0.064
Vitamin E	per µg/MJ	1.00 (1.00, 1.00)	0.100	1.00 (1.00, 1.00)	0.786	0.923
Calcium	per mg/MJ	1.00 (1.00, 1.00)	0.732	1.00 (1.00, 1.00)	0.167	0.188
Iron	per mg/MJ	0.97 (0.94, 1.01)	0.131	0.99 (0.95, 1.04)	0.829	0.127
Zinc	per mg/MJ	1.11 (1.01, 1.22)	0.028	1.21 (1.06, 1.38)	0.005	0.007

95%CI, 95% Confidence Interval; SFAs, saturated fatty acids; PUFAs, polyunsaturated fatty acids; MUFAs, monounsaturated fatty acids.
* Adjusted for age, ethnicity, marital status, socioeconomic status, physical activity, body mass index, smoking status, alcohol consumption, and total energy intake
***P*-interaction represents the statistical significance for interaction item of dietary factor and age where age was modelled linearly in the Cox proportional regression.

Supplementary Table S6 Subgroup analysis by age on associations between nutrient intakes and in-hospital risk of frailty within the UK Women's Cohort Study

		**Risk of Frailty (Hazard Ratio, 95%CI)**				
		**<60 years old**		**≥60 years old**		*P-interaction with age
		Adjusted*	P*	Adjusted*	P*	
Energy intake	per MJ	1.01 (1.00, 1.02)	0.012	1.01 (0.99, 1.02)	0.229	0.127
Protein	per %energy	1.01 (1.00, 1.02)	0.135	1.00 (0.99, 1.02)	0.815	0.319
Carbohydrate	per %energy	1.00 (0.99, 1.00)	0.355	0.99 (0.99, 1.00)	0.082	0.279
Fat	per %energy	1.00 (0.99, 1.01)	0.797	1.01 (1.00, 1.01)	0.165	0.100
SFAs	per %energy	1.00 (0.99, 1.01)	0.676	1.02 (1.00, 1.03)	0.017	0.019
PUFAs	per %energy	1.00 (0.98, 1.02)	0.710	0.98 (0.96, 1.01)	0.259	0.057
MUFAs	per %energy	1.00 (0.99, 1.02)	0.848	1.01 (0.99, 1.03)	0.247	0.249
Vitamin C	per mg/MJ	1.00 (0.99, 1.00)	0.551	1.00 (0.99, 1.00)	0.102	0.914
Vitamin B1	per µg/MJ	1.00 (1.00, 1.00)	0.791	1.00 (1.00, 1.00)	0.077	0.007
Vitamin B2	per µg/MJ	1.00 (1.00, 1.00)	0.619	1.00 (1.00, 1.00)	0.127	0.823
Vitamin B6	per µg/MJ	1.00 (1.00, 1.00)	0.569	1.00 (1.00, 1.00)	0.502	0.154
Vitamin B12	per µg/MJ	1.12 (1.00, 1.25)	0.047	0.98 (0.86, 1.12)	0.780	0.808
Folate	per µg/MJ	1.00 (1.00, 1.00)	0.853	1.00 (0.99, 1.00)	0.380	0.829
Vitamin A	per µg/MJ	1.00 (1.00, 1.00)	0.196	1.00 (1.00, 1.00)	0.422	0.770
Vitamin D	per µg/MJ	1.05 (0.85, 1.28)	0.655	0.99 (0.78, 1.25)	0.914	0.357
Vitamin E	per µg/MJ	1.00 (1.00, 1.00)	0.329	1.00 (1.00, 1.00)	0.210	0.626
Calcium	per mg/MJ	1.00 (1.00, 1.00)	0.060	1.00 (1.00, 1.00)	<0.001	0.087
Iron	per mg/MJ	0.95 (0.90, 1.01)	0.123	0.95 (0.89, 1.02)	0.145	0.496
Zinc	per mg/MJ	1.09 (0.93, 1.27)	0.289	1.05 (0.86, 1.27)	0.648	0.087

95%CI, 95% Confidence Interval; SFAs, saturated fatty acids; PUFAs, polyunsaturated fatty acids; MUFAs, monounsaturated fatty acids.

* Adjusted for age, ethnicity, marital status, socioeconomic status, physical activity, body mass index, smoking status, alcohol consumption, and total energy intake

***P*-interaction represents the statistical significance for interaction item of dietary factor and age where age was modelled linearly in the Cox proportional regression.

Supplementary Table S7 Sensitivity analysis to check for reverse causation on main foods (per 10g/MJ) within the UK Women's Cohort Study

	**Risk of Pre-Frailty or more severe (HR, 95%CI)**				**Risk of Frailty (HR, 95%CI)**			
	Unadjusted	*P*	Adjusted*	*P**	Unadjusted	*P*	Adjusted*	*P**
**Food groups**								
Vegetables	1.00 (0.99, 1.01)	0.691	1.00 (0.99, 1.01)	0.640	0.99 (0.98, 1.01)	0.328	0.99 (0.97, 1.01)	0.290
Fruits	1.01 (1.00, 1.01)	0.047	1.00 (0.99, 1.01)	0.481	1.01 (1.00, 1.03)	0.010	1.00 (0.98, 1.01)	0.534
Total fish	1.16 (1.10, 1.23)	<0.001	1.00 (0.94, 1.06)	0.923	1.20 (1.10, 1.30)	<0.001	0.95 (0.86, 1.05)	0.335
Processed meat	1.69 (1.52, 1.88)	<0.001	1.29 (1.14, 1.46)	<0.001	1.85 (1.57, 2.19)	<0.001	1.30 (1.07, 1.59)	0.010
Red meat	1.25 (1.20, 1.30)	<0.001	1.12 (1.06, 1.17)	<0.001	1.36 (1.27, 1.45)	<0.001	1.17 (1.09, 1.26)	<0.001
Poultry	1.12 (1.04, 1.21)	0.002	1.06 (0.98, 1.16)	0.167	1.07 (0.95, 1.21)	0.252	0.99 (0.87, 1.14)	0.931
Total meat	1.16 (1.12, 1.19)	<0.001	1.07 (1.04, 1.11)	<0.001	1.20 (1.15, 1.25)	<0.001	1.09 (1.04, 1.15)	0.001

HR, Hazards Ratio; 95%CI, 95% Confidence Interval.

*Adjusted for age, ethnicity, marital status, socioeconomic status, physical activity, body mass index, smoking status, alcohol consumption, and total energy intake;

Supplementary Table S8 Sensitivity analysis to check for reverse causation on nutrient intakes within the UK Women's Cohort Study

		Risk of Pre-Frailty or more severe (HR, 95%CI)				Risk of Frailty (HR, 95%CI)			
		Unadjusted	*P*	Adjusted*	*P**	Unadjusted	*P*	Adjusted*	*P**
Energy intake	per MJ	1.00 (1.00, 1.01)	0.322	1.01 (1.00, 1.01)	0.056	1.01 (1.00, 1.02)	0.051	1.01 (1.00, 1.02)	0.008
Protein	per %energy	1.03 (1.02, 1.03)	<0.001	1.01 (1.00, 1.01)	0.028	1.03 (1.02, 1.04)	<0.001	1.00 (0.99, 1.01)	0.950
Carbohydrate	per %energy	1.00 (1.00, 1.00)	0.929	1.00 (1.00, 1.00)	0.178	1.00 (1.00, 1.00)	0.839	1.00 (0.99, 1.00)	0.156
Fat	per %energy	1.00 (1.00, 1.00)	0.276	1.00 (1.00, 1.00)	0.924	1.00 (1.00, 1.00)	0.963	1.00 (1.00, 1.01)	0.315
SFAs	per %energy	1.01 (1.00, 1.01)	0.002	1.00 (1.00, 1.01)	0.243	1.02 (1.01, 1.02)	<0.001	1.01 (1.00, 1.02)	0.059
PUFAs	per %energy	0.97 (0.96, 0.98)	<0.001	1.00 (0.99, 1.01)	0.945	0.96 (0.94, 0.97)	<0.001	1.00 (0.98, 1.01)	0.758
MUFAs	per %energy	0.99 (0.99, 1.00)	0.056	1.00 (0.99, 1.01)	0.968	1.00 (0.98, 1.01)	0.395	1.01 (0.99, 1.02)	0.379
Vitamin C	per mg/MJ	1.00 (1.00, 1.00)	0.084	1.00 (1.00, 1.00)	0.936	1.00 (1.00, 1.01)	0.224	1.00 (0.99, 1.00)	0.448
Vitamin B1	per µg/MJ	1.00 (1.00, 1.00)	0.002	1.00 (1.00, 1.00)	0.805	1.00 (1.00, 1.00)	<0.001	1.00 (1.00, 1.00)	0.543
Vitamin B2	per µg/MJ	1.00 (1.00, 1.00)	<0.001	1.00 (1.00, 1.00)	0.616	1.00 (1.00, 1.00)	0.009	1.00 (1.00, 1.00)	0.061
Vitamin B6	per µg/MJ	1.00 (1.00, 1.00)	<0.001	1.00 (1.00, 1.00)	0.026	1.00 (1.00, 1.00)	0.002	1.00 (1.00, 1.00)	0.439
Vitamin B12	per µg/MJ	1.33 (1.27, 1.41)	<0.001	1.08 (1.02, 1.15)	0.008	1.37 (1.26, 1.49)	<0.001	1.00 (0.91, 1.10)	0.927
Folate	per µg/MJ	1.00 (1.00, 1.01)	<0.001	1.00 (1.00, 1.00)	0.406	1.00 (1.00, 1.01)	0.010	1.00 (1.00, 1.00)	0.653
Vitamin A	per µg/MJ	1.00 (1.00, 1.00)	<0.001	1.00 (1.00, 1.00)	0.023	1.00 (1.00, 1.00)	<0.001	1.00 (1.00, 1.00)	0.831
Vitamin D	per µg/MJ	1.34 (1.23, 1.47)	<0.001	1.08 (0.98, 1.20)	0.138	1.37 (1.19, 1.58)	<0.001	0.99 (0.84, 1.17)	0.878
Vitamin E	per µg/MJ	1.00 (1.00, 1.00)	<0.001	1.00 (1.00, 1.00)	0.875	1.00 (1.00, 1.00)	<0.001	1.00 (1.00, 1.00)	0.681
Calcium	per mg/MJ	1.00 (1.00, 1.00)	0.177	1.00 (1.00, 1.00)	0.379	1.00 (1.00, 1.00)	0.171	1.00 (1.00, 1.00)	<0.001
Iron	per mg/MJ	1.01 (0.98, 1.04)	0.467	0.99 (0.96, 1.02)	0.390	1.00 (0.96, 1.05)	0.992	0.96 (0.91, 1.01)	0.078
Zinc	per mg/MJ	1.37 (1.27, 1.48)	<0.001	1.08 (0.99, 1.18)	0.070	1.43 (1.27, 1.61)	<0.001	1.01 (0.88, 1.15)	0.934

HR, Hazards Ratio; 95%CI, 95% Confidence Interval; SFAs, saturated fatty acids; PUFAs, polyunsaturated fatty acids; MUFAs, monounsaturated fatty acids.

* Adjusted for age, ethnicity, marital status, socioeconomic status, physical activity, body mass index, smoking status, alcohol consumption, and total energy intake

Supplementary Table S9 Sensitivity analysis excluding participants aged <65y at diagnosis on main foods (per 10g/MJ) within the UK Women's Cohort Study

	Risk of Pre-Frailty or more severe (HR, 95%CI)				Risk of Frailty (HR, 95%CI)			
	Unadjusted	*P*	Adjusted*	*P**	Unadjusted	*P*	Adjusted*	*P**
**Food groups**								
Vegetables	0.98 (0.97, 0.99)	0.003	0.99 (0.98, 1.01)	0.396	0.97 (0.95, 0.99)	0.005	0.98 (0.96, 1.01)	0.154
Fruits	1.01 (1.00, 1.02)	0.284	1.00 (0.99, 1.01)	0.765	1.01 (1.00, 1.03)	0.135	1.00 (0.98, 1.01)	0.704
Total fish	1.17 (1.09, 1.25)	<0.001	0.98 (0.90, 1.06)	0.597	1.12 (1.00, 1.24)	0.047	0.89 (0.78, 1.01)	0.075
Processed meat	1.88 (1.63, 2.18)	<0.001	1.30 (1.09, 1.55)	0.004	1.96 (1.58, 2.44)	<0.001	1.34 (1.03, 1.75)	0.030
Red meat	1.33 (1.26, 1.41)	<0.001	1.16 (1.09, 1.24)	<0.001	1.35 (1.24, 1.47)	<0.001	1.17 (1.06, 1.30)	0.002
Poultry	1.17 (1.05, 1.30)	0.004	1.08 (0.96, 1.22)	0.194	1.16 (0.99, 1.36)	0.071	1.08 (0.90, 1.29)	0.396
Total meat	1.22 (1.17, 1.27)	<0.001	1.11 (1.06, 1.16)	<0.001	1.23 (1.16, 1.30)	<0.001	1.11 (1.04, 1.19)	0.002

HR, Hazards Ratio; 95%CI, 95% Confidence Interval.

*Adjusted for age, ethnicity, marital status, socioeconomic status, physical activity, body mass index, smoking status, alcohol consumption, and total energy intake;

Supplementary Table S10 Sensitivity analysis excluding participants aged <65y at diagnosis on nutrient intakes within the UK Women's Cohort Study

		**Risk of Pre-Frailty or more severe (HR, 95%CI)**				**Risk of Frailty (HR, 95%CI)**			
		Unadjusted	*P*	Adjusted*	*P**	Unadjusted	*P*	Adjusted*	*P**
Energy intake	per MJ	1.01 (1.00, 1.02)	0.014	1.00 (1.00, 1.01)	0.315	1.01 (1.00, 1.02)	0.099	1.00 (0.99, 1.02)	0.628
Protein	per %energy	1.03 (1.02, 1.04)	<0.001	1.01 (1.01, 1.03)	0.003	1.03 (1.01, 1.04)	<0.001	1.00 (0.99, 1.02)	0.761
Carbohydrate	per %energy	1.00 (1.00, 1.00)	0.782	1.00 (1.00, 1.00)	0.645	1.00 (0.99, 1.00)	0.606	1.00 (0.99, 1.00)	0.195
Fat	per %energy	1.00 (1.00, 1.00)	0.885	1.00 (0.99, 1.00)	0.231	1.00 (1.00, 1.01)	0.170	1.00 (1.00, 1.01)	0.364
SFAs	per %energy	1.02 (1.01, 1.02)	<0.001	1.00 (0.99, 1.01)	0.990	1.03 (1.02, 1.04)	<0.001	1.01 (1.00, 1.02)	0.040
PUFAs	per %energy	0.95 (0.94, 0.97)	<0.001	0.99 (0.98, 1.01)	0.379	0.94 (0.92, 0.96)	<0.001	0.98 (0.96, 1.01)	0.136
MUFAs	per %energy	0.99 (0.98, 1.00)	0.104	0.99 (0.98, 1.00)	0.245	1.00 (0.98, 1.02)	0.966	1.01 (0.99, 1.02)	0.536
Vitamin C	per mg/MJ	1.00 (1.00, 1.00)	0.384	1.00 (1.00, 1.00)	0.446	1.00 (0.99, 1.00)	0.505	1.00 (0.99, 1.00)	0.303
Vitamin B1	per μg/MJ	1.00 (1.00, 1.00)	0.001	1.00 (1.00, 1.00)	0.140	1.00 (1.00, 1.00)	<0.001	1.00 (1.00, 1.00)	0.106
Vitamin B2	per μg/MJ	1.00 (1.00, 1.00)	<0.001	1.00 (1.00, 1.00)	0.014	1.00 (1.00, 1.00)	0.057	1.00 (1.00, 1.00)	0.242
Vitamin B6	per μg/MJ	1.00 (1.00, 1.00)	<0.001	1.00 (1.00, 1.00)	0.003	1.00 (1.00, 1.00)	0.009	1.00 (1.00, 1.00)	0.679
Vitamin B12	per μg/MJ	1.34 (1.25, 1.43)	<0.001	1.09 (1.01, 1.18)	0.027	1.24 (1.12, 1.38)	<0.001	0.96 (0.85, 1.08)	0.474
Folate	per μg/MJ	1.00 (1.00, 1.01)	<0.001	1.00 (1.00, 1.00)	0.068	1.00 (1.00, 1.01)	0.050	1.00 (1.00, 1.00)	0.743
Vitamin A	per μg/MJ	1.00 (1.00, 1.00)	<0.001	1.00 (1.00, 1.00)	0.482	1.00 (1.00, 1.00)	<0.001	1.00 (1.00, 1.00)	0.385
Vitamin D	per μg/MJ	1.38 (1.24, 1.54)	<0.001	1.11 (0.97, 1.26)	0.122	1.30 (1.10, 1.54)	0.002	0.97 (0.79, 1.19)	0.783
Vitamin E	per μg/MJ	1.00 (1.00, 1.00)	0.001	1.00 (1.00, 1.00)	0.799	1.00 (1.00, 1.00)	0.019	1.00 (1.00, 1.00)	0.718
Calcium	per mg/MJ	1.00 (1.00, 1.00)	0.668	1.00 (1.00, 1.00)	0.576	1.00 (1.00, 1.00)	0.066	1.00 (1.00, 1.00)	0.001
Iron	per mg/MJ	1.05 (1.01, 1.09)	0.019	1.04 (0.99, 1.08)	0.098	1.01 (0.95, 1.07)	0.811	0.98 (0.92, 1.05)	0.620
Zinc	per mg/MJ	1.53 (1.37, 1.70)	<0.001	1.22 (1.09, 1.38)	0.001	1.39 (1.19, 1.63)	<0.001	1.06 (0.89, 1.27)	0.511

HR, Hazards Ratio; 95%CI, 95% Confidence Interval; SFAs, saturated fatty acids; PUFAs, polyunsaturated fatty acids; MUFAs, monounsaturated fatty acids.

* Adjusted for age, ethnicity, marital status, socioeconomic status, physical activity, body mass index, smoking status, alcohol consumption, and total energy intake
